# Hypoglycemic active principles from the leaves of *Bauhinia holophylla*: Comprehensive phytochemical characterization and *in vivo* activity profile

**DOI:** 10.1371/journal.pone.0258016

**Published:** 2021-09-24

**Authors:** Luiz Leonardo Saldanha, Aislan Quintiliano Delgado, Laurence Marcourt, Nathalia Aparecida de Paula Camaforte, Priscilla Maria Ponce Vareda, Samad Nejad Ebrahimi, Wagner Vilegas, Anne Lígia Dokkedal, Emerson Ferreira Queiroz, Jean-Luc Wolfender, José Roberto Bosqueiro

**Affiliations:** 1 Faculty of Sciences, São Paulo State University (UNESP), Bauru, São Paulo, Brazil; 2 School of Pharmaceutical Sciences, University of Geneva, Geneva, Switzerland; 3 Institute of Pharmaceutical Sciences of Western Switzerland (ISPSO), University of Geneva, Geneva, Switzerland; 4 Department of Phytochemistry, Medicinal Plants and Drugs Research Institute, Shahid Beheshti University, G. C., Evin, Tehran, Iran; 5 Institute of Biociences, São Paulo State University (UNESP), São Vicente, São Paulo, Brazil; Cairo University, EGYPT

## Abstract

*Bauhinia holophylla* leaves, also known as "pata-de-vaca", are traditionally used in Brazil to treat diabetes. Although the hypoglycemic activity of this medicinal plant has already been described, the active compounds responsible for the hypoglycemic activity have not yet been identified. To rapidly obtain two fractions in large amounts compatible with further *in vivo* assay, the hydroalcoholic extract of *B*. *holophylla* leaves was fractionated by Vacuum Liquid Chromatography and then purified by medium pressure liquid chromatography combined with an *in vivo* Glucose Tolerance Test in diabetic mice. This approach resulted in the identification of eleven compounds (**1**–**11**), including an original non-cyanogenic cyanoglucoside derivative. The structures of the isolated compounds were elucidated by nuclear magnetic resonance and high-resolution mass spectrometry. One of the major compounds of the leaves, lithospermoside (**3**), exhibited strong hypoglycemic activity in diabetic mice at the doses of 10 and 20 mg/kg b.w. and prevents body weight loss. The proton nuclear magnetic resonance (^1^H NMR) quantification revealed that the hydroalcoholic leaves extract contained 1.7% of lithospermoside (**3**) and 3.1% of flavonoids. The NMR analysis also revealed the presence of a high amount of pinitol (**4**) (9.5%), a known compound possessing *in vivo* hypoglycemic activity. The hypoglycemic properties of the hydroalcoholic leaves extract and the traditional water infusion extracts of the leaves of *B*. *holophylla* seem thus to be the result of the activity of three unrelated classes of compounds. Such results support to some extent the traditional use of *Bauhinia holophylla* to treat diabetes.

## Introduction

*Bauhinia holophylla* (Bong.) Steud. belongs to Fabaceae and is a shrub naturally occurring in the Brazilian "Cerrado". The *Bauhinia* L. genus consists of *ca*. 300–350 species distributed in most tropical regions, including Africa, Asia and South America, popularly known as "pata-de-vaca" or "cow’s paw" because of the bilobed leaf morphology [1]. The leaves of *B*. *holophylla* are popularly used as a decoction for the treatment of *Diabetes mellitus* (DM) [2]. Recently, the hydroalcoholic extract of the leaves of this plant was reported to reduce blood glucose in diabetic animals using an intraperitoneal glucose tolerance test (ipGTT) [3]. The ipGTT test is used in clinical practices to detect disorders in glucose metabolism linked to human diseases such as diabetes or metabolic syndrome [4]. This assay follows the blood glucose clearance after the glucose loading by intraperitoneal injection [4]. The glucose-lowering effect of this crude extract was found to be related to the glycogen synthase kinase 3 beta (GSK3-β) inhibition and glycogenesis activation [3].

There have been several other studies showing the anti-diabetic activity of the aqueous and hydroalcoholic leaves extracts of *Bauhinia* species used as hypoglycemic tea to treat DM [1]. The leaves and the stem barks of any *Bauhinia* have long been used nearly indiscriminately in Brazilian folk medicine as these species are often confused due to their morphological similarity. Although in Brazil *Bauhinia forficata* Link is considered the authentic "pata-de-vaca" possessing hypoglycemic effects and the most investigated species [1, 5]. Recently, *B*. *forficata* extract has been shown to be effective in minimizing the deleterious effects of bisphenol A on blood glucose hepatic antioxidant status and glycogen store capacity in Wistar rats [6].

Besides flavonoids, other constituents such as alkaloids, aromatic acids, bibenzyls, terpenes, steroids, and tannins were also reported from other *Bauhinia* species [7]. Despite the extensive phytochemical and biological investigations of these species, there is still a lack of conclusive studies that identify the active principle using *in vivo* models [7].

In the present study, the hydroalcoholic extract *B*. *holophylla* was fractionated to obtain coarse fractions exhibiting a very complementary chemical composition assessed by nuclear magnetic resonance (NMR) and liquid chromatography coupled to mass spectrometry (LC-MS) metabolite profiling. The extract purification allowed the identification of eleven compounds (**1**–**11**), including an original non-cyanogenic cyanoglucoside derivative, isolated for the first time from *B*. *holophylla*. The extract, the coarse fractions, and the non-cyanogenic cyanoglucoside derivative, unreported for its anti-diabetic properties, were assessed by the *in vivo* intraperitoneal glucose tolerance test (ipGTT).

## Materials and methods

### General experimental procedures

The optical rotations were measured in methanol solutions on a JASCO polarimeter in a 1 cm tube. The electronic circular dichroism (ECD) spectra were recorded in MeOH (Sigma-Aldrich^®^—St. Louis, MO, USA) with a Jasco F-815 spectrometer. The nuclear magnetic resonance (NMR) spectroscopic data were recorded on a 500 MHz Varian Inova spectrometer and on a Bruker Avance III HD 600 MHz NMR spectrometer equipped with a QCI 5 mm Cryoprobe and a SampleJet automated sample changer (Bruker BioSpin, Rheinstetten, Germany). Chemical shifts are reported in parts per million (*δ*) using the residual D_2_O (99.90% D, Eurisotop) signal (*δ*_H_ 4.81) or DMSO-*d*_6_ (99.80% D, Eurisotop) (*δ*_H_ 2.50) as internal standards for ^1^H and coupling constants (*J*) are reported in Hz. Complete assignments were performed based on 2D-NMR experiments (COSY, NOESY, HSQC, and HMBC). Electrospray ionization coupled to high-resolution mass spectrometry (ESI-HRMS) analyses were conducted on a Micromass LCT Premier time-of-flight mass spectrometer from Waters with an ESI interface (Waters, Milford, MA, USA). ESI-MS/MS data were obtained on an Accela LCQ Fleet mass spectrometer with ion trap 3D and electrospray ionization from Thermo Scientific with flow injection analysis (FIA) using a syringe pump (Thermo Scientific, San Jose, CA, USA). All used organic solvents in chromatographic procedures were high performance liquid chromatography (HPLC) grade while H_2_O was purified using a Milli-Q system. EtOH used for extraction was purchased from Sigma-Aldrich^®^—St. Louis, MO, USA.

### Plant material

Samples of *Bauhinia holophylla* (Bong.) Steud. leaves were collected in November 2018 at the Botanical Garden of Bauru (22°20′30′′S and 49°00′30′′W), SP, Brazil. The plant name was verified with http://www.theplantlist.org. Voucher specimens were identified by Dr. Ângela Maria Studart da Fonseca Vaz and stored at the Herbarium of the Botanical Garden of Rio de Janeiro (Rio de Janeiro/RJ, Brazil) under code number RB 507.043. Fresh leaves were dried at 40°C for 48 h. The access and shipment of a component of genetic heritage, as issued by the National Council for Scientific and Technological Development (Conselho Nacional de Desenvolvimento Científico e Tecnológico–CNPq), was performed under authorization No. 010468/2014-51 of Genetic Heritage Management Council (Conselho de Gestão do Patrimônio Genético–CGEN).

### Extraction

Powdered leaves of *B*. *holophylla* (300 g) were extracted with EtOH: H_2_O 7:3% v/v by percolation at room temperature [8]. The filtrate was concentrated to dryness under reduced pressure at 40°C yielding the hydroalcoholic leaves extract (29.5% dry weight). The powdered leaves of *B*. *holophylla* (0.99 g) were also extracted by water infusion (traditional preparation) with 100 mL of water for 30 min. The solution was filtrated, frozen, and lyophilized affording 0.19 g (19.9%) of the water infusion extract.

### HPLC-PDA-ELSD analyses

High performance liquid chromatography coupled to photodiode array and evaporative light scattering detectors (HPLC-PDA-ELSD) analyses were conducted on an HP 1100 system equipped with a PAD (Agilent Technologies, Santa Clara, CA, USA) connected to an ELSD Sedex 85 (Sedere, Oliver, France). Separations were achieved using an X-Bridge C_18_ column (250 × 4.6 mm i.d., 5 μm, Waters, Milford, MA, USA) and the mobile phase was composed of MeOH (B) and H_2_O (A), both containing 0.1% formic acid. The separation was performed in gradient mode as follows: 5% to 100% of B in 60 min. Flow rate was 1 mL/min; injection volume was 10 μL; sample concentration 10 mg/mL in the mobile phase. The UV absorbance was measured at 254 and 366 nm and the UV-PDA spectra were recorded between 190 and 600 nm (step 2 nm). The ELSD detection parameters were pressure 3.5 bar, 45°C, split to provide a 500 μL/min flow rate, gain 8. This method was used for the profiling of the main components present in the crude extract.

### UHPLC-TOF-HRMS analyses

High-resolution mass spectrometry (HRMS) metabolite profiling of the extract, fractions, and pure products was performed on a Micromass-LCT Premier Time of Flight (TOF) mass spectrometer (Waters) equipped with an electrospray interface and coupled to an Acquity ultra-.performance liquid chromatography (UPLC) system (Waters) using a generic method previously described [9].

### Fractionation of the hydroalcoholic leaves extract using vacuum liquid chromatography (VLC)

The hydroalcoholic leaves extract (9 g) was fractionated by VLC in a sintered glass filter funnel (250 mL) dry-packed with Zeoprep 60 C_18_ as the stationary phase (40−63 μm, Zeochem, Uetikon am See, Switzerland). The extract was eluted in two steps; the first step was performed with a mixture of MeOH: H_2_O (+0.1% formic acid) 3:7 v/v (3.75 L), followed by a second elution step achieved with a mixture of MeOH: H_2_O (+0.1% formic acid) 8:2 v/v (1.75 L). Two fractions were obtained, polar fraction 30% (4.1 g) (PF) and flavonoid fraction 80% (1.1 g) (FF), respectively. The preparative VLC conditions were optimized using an analytical solid-phase extraction cartridge packed with the same stationary phase.

### Purification of the hydroalcoholic leaves extract

The hydroalcoholic leaves extract (12 g) was fractionated by medium pressure liquid chromatography (MPLC) using Zeoprep C_18_ as the stationary phase (460 × 49 mm i.d., 15−25 μm, Zeochem, Uetikon am See, Switzerland) using gradient elution with H_2_O (A) and MeOH (B) acidified with 0.1% of formic acid at 35°C. The column was first conditioned with 5% of B over 56 minutes followed by 5–33% of B over 9:40h; 33–60% of B over 6:40h; 60–100% 4:34h and 100% of B for 3:37h. These conditions were first optimized on an analytical HPLC column (250 × 4.6 mm i.d., 15−25 μm, Zeochem, Uetikon am See, Switzerland) packed with the same stationary phase and then geometrically transferred to the preparative scale [10]. The hydroalcoholic extract was introduced into the MPLC column by dry injection by mixing 12 g of the extract with 36 g of the Zeoprep C18 stationary phase (40−63 μm, Zeochem, Uetikon am See, Switzerland). The mixture was conditioned in a dry-load cell (11.5 × 2.7 cm i.d.). The dry-load cell was connected subsequently between the pumps and the MPLC column. The flow rate was set to 12 mL/min, and UV detection was performed at 254 nm. MPLC separation yielded 98 fractions, which were analyzed by UHPLC-UV-ELSD. Fractions F3 yielded compound **4** (2.3 g), F4 yielded compound **1** (11 mg), F5 yielded compound **2** (7 mg), F6 yielded compound **3** (244 mg), and fraction F61 yielded compound **7** (8.8 mg). The other MPLC fractions containing mixtures of flavonoids were combined according to the similarity of their composition and subjected to purification by semi-preparative HPLC coupled to Refractive Index detector (RI) (Knauer^®^ 2300, Berlin, Germany) using a C_18_ (250 × 10.0 mm i.d.; 5 μm) column and a mixture of MeOH-H_2_O (1:1) acidified with 0.1% of formic acid as the mobile phase. Using these conditions fractions F57-58 (82 mg) were combined and purified affording compounds **5** (21.9 mg) and **6** (18.9 mg). Fractions F64-69 (96 mg) were combined and purified affording compounds **8** (8.0 mg) and **9** (9.4 mg), while fractions F79-84 afforded compounds **10** (13.0 mg) and **11** (1.7 mg).

### Identification of the isolated compound

(-)-(1E,4*R*,5*S*,6*S*)-6-(*β*-D-glucopyranosyloxy)-4,5-dihydroxy-2-cyclohexen-1-ylideneacetonitrile **(1)**: Amorphous white powder; UV (MeOH) λ_max_: 269 nm; [α]_D_21–11.95° (c 0.5 MeOH); ECD (MeOH, c 0.1 cm) [*θ*]_296_ +492, [*θ*]_272_−1989, *θ*]_251_ +365, [*θ*]_232_−9983 (S1 Fig in S1 File); ^1^H NMR (D_2_O, 499.9 MHz) δ 3.45 (1H, m, H-2’), 3.46 (2H, m, H-4’, H-5’), 3.54 (1H, t, *J* = 9.1 Hz, H-3’), 3.74 (1H, ddd, *J* = 10.5, 8.3, 2.0 Hz, H-5), 3.75 (1H, d, *J* = 12.5 Hz, H-6’b), 3.89 (1H, dd, *J* = 12.5, 1.6 Hz, H-6’a), 4.42 (1H, dt, *J* = 8.3, 2.0 Hz, H-4), 4.62 (1H, dd, *J* = 10.5, 2.0 Hz, H-6), 4.79 (1H, d, *J* = 7.8 Hz, H-1’), 5.85 (1H, t, *J* = 2.0 Hz, H-7), 6.19 (1H, dt, *J* = 10.1, 2.0 Hz, H-3), 6.65 (1H, dd, *J* = 10.1, 2.3 Hz, H-2); ^13^C NMR (D_2_O. 125.7 MHz) 63.3 (C-6’), 72.3 (C-4’), 74.3 (C-4), 76.4 (C-2’), 78.6 (C-3’), 78.8 (C-5’), 79.1 (C-5), 82.1 (C-6), 96.9 (C-7), 106.2 (C-1’), 126.6 (C-2), 141.3 (C-3), 160.0 (C-1).; ESI-HRMS: *m/z* 328.1055 [M-H]^-^ (calcd for C_14_H_18_NO_8_, 328.1032).

(+)-(1E,4*R*,5*S*,6*S*)-4,5,6-trihydroxy-2-cyclohexen-1-ylideneacetonitrile **(2)**: Amorphous white powder; UV (MeOH) λ_max_: 269 nm; [α]_D_^21^ +7.48° (c 0.1 MeOH); ECD (MeOH, c 0.1 cm) [*θ*]_280_−1589, [*θ*]_227_ +23912 [*θ*]_206_−11647 (S1 Fig in S1 File); ^1^H NMR (D_2_O, 500 MHz) δ 3.51 (1H, dd, *J* = 10.4, 8.1 Hz, H-5), 4.31 (1H, dd, *J* = 10.4, 2.0 Hz, H-6), 4.37 (1H, dt, *J* = 8.1, 2.0 Hz, H-4), 5.65 (1H, d, *J* = 2.0 Hz, H-7), 6.18 (1H, dd, *J* = 10.1, 2.0 Hz, H-3), 6.65 (1H, dd, *J* = 10.1, 2.0 Hz, H-2); ^13^C NMR (D_2_O. 125.7 MHz) 74.5 (C-6), 74.6 (C-4), 79.6 (C-5), 95.2 (C-7), 120.7 (C-8), 126.6 (C-2), 141.7 (C-3), 162.4 (C-1); ESI-HRMS *m/z* 168.0694 [M+H]^+^ (calcd for C_8_H_10_NO_3_, 168.0661).

(-)-Lithospermoside **(3)**: Amorphous white powder; UV (MeOH) λ_max_: 269 nm; [α]_D_21–209.12° (c 0.1 MeOH); ECD (MeOH, c 0.1 cm) [*θ*]_266_−8664, [*θ*]_232_ +29488, *θ*]_206_−6981 (S1 Fig in S1 File); ^¹^H NMR (D_2_O, 499.9 MHz) 3.44 (1H, m, H-4’), 3.45 (1H, m, H-5’), 3.52 (2H, m, H-2’, H-3’), 3.75 (1H, dd, *J* = 12.4, 5.4 Hz, H-6’b), 3.93 (1H, dd, *J* = 12.4, 2.1 Hz, H-6’a), 3.97 (1H, dd, *J* = 8.2, 6.1 Hz, H-5), 4.31 (1H, dt, *J* = 6.1, 3.2, 1.7 Hz, H-4), 4.86 (1H, dd, *J* = 8.2, 1.7 Hz, H-6), 4.90 (1H, d, *J* = 7.8 Hz, H-1’), 5.65 (1H, t, *J* = 3.2, 1.7 Hz, H-6), 6.14 (1H, dd, *J* = 10.2, 3.2 Hz, H-3), 6.36 (1H, dd, *J* = 10.2, 1.8 Hz, H-2); ¹³C NMR (D_2_O, 125.7 MHz) 63.7 (C-6’), 72.5 (C-4’), 72.7 (C-4), 75.6 (C-2’), 76.7 (C-5), 78.6 (C-6), 78.7 (C-3’), 78.9 (C-5’), 99.9 (C-7), 102.6 (C-1’), 120.5 (CN), 129.8 (C-2), 139.0 (C-3), 158.1 (C-1); ESI- HRMS: *m/z* 328.1059 [M-H]^-^ (calcd for C_14_H_18_NO_8_, 328.1032).

Pinitol (**4**): ^1^H NMR (D_2_O, 600 MHz) δ 3.35 (1H, t, *J* = 9.7 Hz, H-3), 3.60 (3H, s, OCH_3_), 3.66 (1H, t, *J* = 9.7 Hz, H-2), 3.77 (1H, dd, *J* = 9.7, 2.9 Hz, H-1), 3.82 (1H, dd, *J* = 9.7, 2.9 Hz, H-4), 4.01 (2H, m, H-5, H-6); ^13^C NMR (D_2_O, 151 MHz) δ 62.7 (OCH_3_), 72.7 (C-4), 73.4 (C-1), 74.5 (C-5, C-6), 75.0 (C-2), 85.7 (C-3).

Myricitrin (**5**): Amorphous yellow powder; UV (MeOH) λ_max_: 350, 255 nm; ^1^H NMR (DMSO-*d*_6_, 499 MHz) δ 0.84 (3H, d, *J* = 6.2 Hz, H-6’’), 3.15 (1H, t, *J* = 9.4 Hz, H-4’’), 3.35 (1H, dq, *J* = 9.4, 6.2 Hz, H-5’’), 3.55 (1H, dd, *J* = 9.4, 3.4 Hz, H-3’’), 3.98 (1H, t, *J* = 3.4, 1.7 Hz, H-2’’), 5.20 (1H, d, *J* = 1.8 Hz, H-1’’), 6.19 (1H, d, *J* = 2.0 Hz, H-6), 6.37 (1H, d, *J* = 2.0 Hz, H-8), 6.88 (2H, s, H-2’, H-6’); ^13^C NMR (DMSO, 126 MHz) δ 17.6 (C-6’’), 70.1 (C-2’’), 70.5 (C-3’’), 70.7 (C-5’’), 71.4 (C-4’’), 93.7 (C-8), 98.8 (C-6), 102.0 (C-1’’), 104.1 (C-10), 108.0 (C-2’, C-6’), 119.7 (C-1’), 134.4 (C-3), 136.6 (C-4’), 145.9 (C-3’, C-5’), 156.5 (C-9), 157.6 (C-2), 161.4 (C-5), 164.4 (C-7), 177.9 (C-4); ESI-HRMS: *m/z* 463.0914 [M-H]^-^ (calcd for C_21_H_19_O_12_, 463.0877); ESI-MS/MS: *m/z* 463 [M-H]^-^, *m/z* 316 [M-147-H]^-^.

Hyperin (**6**): Amorphous yellow powder; UV (MeOH) λ_max_: 354, 303 sh, 255 nm; ^1^H NMR (DMSO-*d*_6_, 499 MHz) δ 3.31 (2H, m, H-5’’, H-6’’b), 3.37 (1H, dd, *J* = 9.6, 3.4 Hz, H-3’’), 3.46 (1H, dd, *J* = 10.0, 5.6 Hz, H-6’’a), 3.57 (1H, dd, *J* = 9.6, 7.7 Hz, H-2’’), 3.65 (1H, d, *J* = 3.4 Hz, H-4’’), 5.37 (1H, d, *J* = 7.7 Hz, H-1’’), 6.19 (1H, d, *J* = 2.0 Hz, H-6), 6.40 (1H, d, *J* = 2.0 Hz, H-8), 6.82 (1H, d, *J* = 8.5 Hz, H-5’), 7.53 (1H, d, *J* = 2.3 Hz, H-2’), 7.66 (1H, dd, *J* = 8.5, 2.3 Hz, H-6’); ^13^C NMR (DMSO-*d*_6_, 126 MHz) δ 60.2 (C-6’’), 68.0 (C-4’’), 71.2 (C-2’’), 73.2 (C-3’’), 75.9 (C-5’’), 93.6 (C-8), 98.8 (C-6), 101.9 (C-1’’), 103.9 (C-10), 115.2 (C-5’), 116.0 (C-2’), 121.1 (C-1’), 122.0 (C-6’), 133.5 (C-3), 144.9 (C-3’), 148.5 (C-4’), 156.3 (C-9), 156.4 (C-2), 161.2 (C-5), 164.3 (C-7), 177.5 (C-4); ESI-HRMS: *m/z* 463.0928 [M-H]^-^ (calcd for C_21_H_19_O_12_, 463.0877); ESI-MS/MS: *m/z* 463 [M-H]^-^, *m/z* 301 [M-162-H]^-^.

Reynoutrin (**7**): Amorphous yellow powder; UV (MeOH) λ_max_: 355, 255 nm. ^1^H NMR (DMSO-*d*_6_, 499 MHz) δ 2.96 (1H, dd, *J* = 11.5, 9.5 Hz, H-5’’b), 3.19 (1H, t, *J* = 8.6 Hz, H-3’’), 3.31 (1H, t, *J* = 8.6, 7.2 Hz, H-2’’), 3.32 (1H, td, *J* = 9.5, 8.6, 5.2 Hz, H-4’’), 3.63 (1H, dd, *J* = 11.5, 5.2 Hz, H-5’’a), 5.33 (1H, d, *J* = 7.2 Hz, H-1’’), 6.16 (1H, d, *J* = 2.0 Hz, H-6), 6.37 (1H, d, *J* = 2.1 Hz, H-8), 6.84 (1H, d, *J* = 8.4 Hz, H-5’), 7.53 (1H, dd, *J* = 8.4, 2.2 Hz, H-6’), 7.56 (1H, d, *J* = 2.2 Hz, H-2’); ^13^C NMR (DMSO-*d*_6_, 126 MHz) δ 65.8 (C-5’’), 69.1 (C-4’’), 73.4 (C-2’’), 75.7 (C-3’’), 93.5 (C-8), 98.8 (C-6), 101.6 (C-1’’), 103.6 (C-10), 115.1 (C-5’), 115.7 (C-2’), 121.2 (C-1’, C-6’), 133.4 (C-3), 145.1 (C-3’), 147.6 (C-4’), 156.4 (C-2, C-9), 161.4 (C-5), 165.4 (C-7), 177.6 (C-4); ESI-HRMS: *m/z* 433.0776 [M-H]^-^ (calcd for C_20_H_17_O_11_, 433.0771); ESI-MS/MS: *m/z* 433 [M-H]^-^, *m/z* 301 [M-132-H]^-^.

Quercetin-3-*O-α-L*-arabinofuranoside (**8**): Amorphous yellow powder; UV (MeOH) λ_max_: 354, 256 nm; ^1^H NMR (DMSO-*d*_6_, 499 MHz) δ 3.28 (1H, dd, *J* = 12.0, 5.1 Hz, H-5’’b), 3.33 (1H, dd, *J* = 12.0, 3.6 Hz, H-5’’a), 3.57 (1H, td, *J* = 6.3, 5.1, 3.6 Hz, H-4’’), 3.73 (1H, dd, *J* = 6.3, 3.9 Hz, H-3’’), 4.16 (1H, dd, *J* = 3.9, 1.4 Hz, H-2’’), 5.59 (1H, d, *J* = 1.4 Hz, H-1’’), 6.20 (1H, d, *J* = 2.0 Hz, H-6), 6.41 (1H, d, *J* = 2.1 Hz, H-8), 6.85 (1H, d, *J* = 8.4 Hz, H-5’), 7.48 (1H, d, *J* = 2.2 Hz, H-2’), 7.55 (1H, dd, *J* = 8.4, 2.2 Hz, H-6’); ^13^C NMR (DMSO-*d*_6_, 126 MHz) δ 60.7 (C-5’’), 77.0 (C-3’’), 82.1 (C-2’’), 85.9 (C-4’’), 93.6 (C-8), 98.7 (C-6), 103.9 (C-10), 107.9 (C-1’’), 115.5 (C-2’), 115.6 (C-5’), 121.0 (C-1’), 121.7 (C-6’), 133.4 (C-3), 145.1 (C-3’), 148.5 (C-4’), 156.4 (C-9), 156.9 (C-2), 161.2 (C-5), 164.4 (C-7), 177.7 (C-4); ESI-HRMS: *m/z* 433.0776 [M-H]^-^ (calcd for C_21_H_19_O_12_, 433.0771); ESI-MS/MS: *m/z* 433 [M-H]^-^, *m/z* 301 [M-132-H]^-^.

Quercitrin (**9**): Amorphous yellow powder; UV (MeOH) λ_max_: 350, 256 nm; ^1^H NMR (DMSO-*d*_6_, 499 MHz) δ 0.81 (3H, d, *J* = 6.0 Hz, H-6’’), 3.14 (1H, t, *J* = 9.4 Hz, H-4’’), 3.21 (1H, dq, *J* = 9.4, 6.0 Hz, H-5’’), 3.51 (1H, dd, *J* = 9.1, 3.3 Hz, H-3’’), 3.98 (1H, dd, *J* = 3.3, 1.6 Hz, H-2’’), 5.25 (1H, d, *J* = 1.6 Hz, H-1’’), 6.19 (1H, d, *J* = 2.0 Hz, H-6), 6.38 (1H, d, *J* = 2.0 Hz, H-8), 6.86 (1H, d, *J* = 8.3 Hz, H-5’), 7.25 (1H, dd, *J* = 8.3, 2.2 Hz, H-6’), 7.29 (1H, d, *J* = 2.2 Hz, H-2’); ^13^C NMR (DMSO, 126 MHz) δ 17.6 (C-6’’), 70.2 (C-2’’), 70.5 (C-3’’), 70.7 (C-5’’), 71.3 (C-4’’), 93.8 (C-8), 98.9 (C-6), 101.9 (C-1’’), 104.1 (C-10), 115.6 (C-5’), 115.7 (C-2’), 120.8 (C-1’), 121.2 (C-6’), 134.3 (C-3), 145.3 (C-3’), 148.6 (C-4’), 156.6 (C-9), 157.4 (C-2), 161.4 (C-5), 164.6 (C-7), 177.8 (C-4); ESI-HRMS: *m/z* 447.0940 [M-H]^-^ (calcd for C_21_H_19_O_11_, 447.0927); ESI-MS/MS: *m/z* 447 [M-H]^-^, *m/z* 301 [M-146-H]^-^.

Quercetin (**10**): Amorphous yellow powder; UV (MeOH) λ_max_: 371, 309sh, 255, nm; ^1^H NMR (MeOD, 499 MHz) δ 6.19 (1H, d, *J* = 2.1 Hz, H-6), 6.39 (1H, d, *J* = 2.1 Hz, H-8), 6.89 (1H, d, *J* = 8.5 Hz, H-5’), 7.63 (1H, dd, *J* = 8.5, 2.2 Hz, H-6’), 7.73 (1H, d, *J* = 2.1 Hz, H-2’); ^13^C NMR (DMSO-*d*_6_, 126 MHz) δ 94.4 (C-8), 99.2 (C-6), 104.5 (C-10), 116.0 (C-2’), 116.2 (C-5’), 121.7 (C-6’), 124.1 (C-1’), 137.2 (C-3), 146.2 (C-3’), 148.0 (C-2), 148.8 (C-4’), 158.2 (C-9), 162.5 (C-5), 165.6 (C-7), 177.3 (C-4); ESI-HRMS: *m/z* 301.0309 [M-H]^-^ (calcd for C_15_H_9_O_7_, 301.0348); ESI-MS: *m/z* 301 [M-H]^-^.

Luteolin (**11)**: Amorphous yellow powder; UV (MeOH) λ_max_: 350, 293sh, 265 nm; ^1^H NMR (DMSO-*d*_6_, 499 MHz) δ 6.18 (1H, d, *J* = 2.1 Hz, H-6), 6.44 (1H, d, *J* = 2.1 Hz, H-8), 6.66 (1H, s, H-3), 6.88 (1H, d, *J* = 8.3 Hz, H-5’), 7.39 (1H, d, *J* = 2.3 Hz, H-2’), 7.41 (1H, dd, *J* = 8.3, 2.3 Hz, H-6’); ESI-HRMS: *m/z* 285.0437 [M-H]^-^ (calcd for C_15_H_9_O_6_, 285.0399); ^13^C NMR (DMSO-*d*_6_, 126 MHz) δ 93.8 (C-8), 98.8 (C-6), 102.9 (C-3), 103.7 (C-10), 113.4 (C-2’), 116.0 (C-5’), 119.0 (C-6’), 121.5 (C-1’), 145.7 (C-3’), 149.7 (C-4’), 157.3 (C-9), 161.5 (C-5), 163.9 (C-2), 164.1 (C-7), 181.6 (C-4); ESI-MS/MS: *m/z* 285 [M-H]^-^.

### Computational methods

Conformational analyses for E/Z isomer of **1**, **2**, and **3** were performed with MacroModel 9.1 software (Schrödinger LLC, New York) using the Optimized Potential for Liquid Simulations 3 (OPLS3) force field in H_2_O. Conformers occurring within a 1.0 kcal/mol energy window from the global minimum were chosen for geometrical optimization and energy calculation using DFT/cam-B3LYP/6-31G** was conducted in MeOH using the SCRF method, with the CPCM model with the Gaussian 09 program [11]. The vibrational analysis was done at the same level to confirm minima. The ECD spectra calculated using TD-DFT/cam-B3LYP/6-31G** in MeOH. The ECD curves were constructed based on rotatory strength dipole velocity (R_*vel*_), and dipole length (R_*len*_) were calculated with a half-band of 0.25 eV using SpecDis v1.61 [12].

### General procedure for NMR quantification

The quantification of lithospermoside (**3**), total flavonoids or pinitol (**4**) was performed by proton nuclear magnetic resonance (^1^H NMR) using TSP (3-(trimethylsilyl)propionic-2,2,3,3-d_4_ acid sodium) as internal standard. A precisely weighed amount the samples were dissolved in 600 μL of an aqueous solution buffered at pH 7 (KH_2_PO_4_) containing a known amount of TSP. ^1^H NMR were recorded at 298 K or 300 K on a 500 or 600 MHz spectrometer (for equipment details see General experimental procedures section). A solvent suppression pulse sequence (noesygppr1d) was used with the following parameters: 64 scans, a pulse width calibrated for 90° flip angle, an automatic receiver gain adjustment, a relaxation delay of 4s, an acquisition time of 1.84s, and 32k data points collected with a sweep width of 65536 Hz. Data processing was performed using the MNova 14.1 software. The raw fid was zero-filled to 64k data points before exponential multiplication with a line broadening factor of 0.3 and Fourier transformation. The spectrum phase was done manually, and a 3^rd^ order polynomial fit baseline correction was applied. Then the signals of interest were peak picking with the GSD (Global Spectral Deconvolution) method analysis and integrated.

### Quantification of lithospermoside (3) by NMR

The quantification of lithospermoside (**3**) in the hydroalcoholic leaves extract was performed by ^1^H NMR [13, 14]. For this, 10.0 mg of hydroalcoholic leaves extract were dissolved in 600 uL of a deuterated phosphate buffer solution (KH_2_PO_4_, pH 7 in D_2_O) containing 3.87 mM of TSP (3-(trimethylsilyl)propionic-2,2,3,3-d_4_ acid sodium). The phosphate buffer was used to avoid variation of proton chemical shifts. The TSP was used as an internal reference standard for chemical shift calibration and for quantification of the active compound in the extract. The signals at δ_H_ 6.35 (1H, dd *J* = 10.2, 1.8, H-2), 6.14 (1H, dd *J* = 10.2, 3.2, H-14) and 5.64 (1H, s, H-7) were used for the quantification of lithospermoside (**3**) after having checked that these multiplets were exactly superimposed to those of pure lithospermoside recorded in the same conditions. Using this method, the quantity of lithospermoside was estimated to be 1.7% w/w in the hydroalcoholic leaves extract. The quantification of lithospermoside in the polar fraction was performed in the same way on a sample (0.9 mg) containing 0.9 mM of TSP. It was estimated to be 5.9% w/w in the polar fraction.

### Quantification of total flavonoids by NMR

The quantification of total flavonoids in the hydroalcoholic leaves extract was performed by ^1^H NMR profiling in D_2_O of the flavonoid fraction taking into consideration its proportion in the extract. The signals around δ_H_ 6.31 and 6.14, usually assigned to H-6 and H-8 protons of flavonoids, have been used for quantification. A known amount of TSP (0.9 mM) was added to this fraction (0.9 mg) and used as an internal standard for calibration. The mean molecular weight of total flavonoids was estimated to 428 g/mol considering the molecular weight and amount of isolated flavonoids. Using these data, the total amount of flavonoids was estimated to 27.5% in the flavonoid fraction and 3.1% w/w in the hydroalcoholic leaf extract of *B*. *holophylla*.

### Quantification of pinitol (4) by NMR

The quantification of pinitol in the hydroalcoholic leaves extract and in the water infusion was performed by ^1^H NMR in D_2_O. The signals at δ_H_ 4.02 (2H, m, H-5, H-6), 3.84 (1H, dd, *J* = 9.9, 2.9 Hz, H-4), 3.78 (1H, dd, *J* = 9.9, 2.9 Hz, H-1) and 3.67 (1H, t, *J* = 9.6 Hz, H-2) have been used for quantification. A known amount of TSP was added to the hydroalcoholic leaves extract (3.87 mM of TSP for 10.0 mg) and in the water infusion (5.81 mM of TSP for 4.8 mg) and used as an internal standard for calibration. Using these data, the amount of pinitol was estimated to 9.5% w/w in the hydroalcoholic extract and 14.3% in the traditional water infusion of *B*. *holophylla* leaves. The quantification of pinitol in the polar fraction (1.5 mg) was performed in the same way on a sample containing 0.9 mM of TSP. It was estimated to be 3.7% w/w in the polar fraction.

### Animals

The experimental protocol was approved by the Animal Experimentation Ethics Committee of UNESP/Botucatu (Process n° 520-CEUA) to Prof. Bosqueiro in 2015). Male Swiss mice (approx. 60 days, weighing 40–50 g) were obtained from the UNESP animal care unit at the campus of the University of Botucatu, and maintained at 22 ± 2°C on a 12-h light-dark cycle (lights on at 6:00 am, lights off at 6:00 pm). Animals were fed with industrialized food (Labina^®^, Purina, Brazil) and water *ad libitum*.

### Induction of experimental diabetes

The induction of diabetes was performed using a single injection of 150 mg/kg b.w. streptozotocin (STZ—Sigma-Aldrich^®^—St. Louis, MO, USA) in mice fasted for 12–14 h. The STZ was dissolved in citrate buffer (pH 4.5) and immediately injected intraperitoneally. The animals were kept fasting for 3 h after induction and for the next 24 h received a 3% glucose solution to prevent hypoglycemia. On the 7^th-^day -post-STZ-injection, the animals that showed fasting glycemia higher than 250 mg/dL associated with polyuria and polydipsia were considered diabetic and included in the study [15].

### Intraperitoneal glucose tolerance test (ipGTT) following *B*. *holophylla* extract and fractions treatment

The animals were randomly divided into eight groups (n = 8/group): CTLSAL—non-diabetic control mice treated with vehicle (0.9% NaCl); STZSAL—diabetic mice treated with vehicle (0.9% NaCl); EXTRACT—diabetic mice treated with hydroalcoholic leaves extract at a dose of 400 mg/kg b.w.; FF50—diabetic mice treated with flavonoid fraction at a dose of 50 mg/kg b.w.; FF100—diabetic mice treated with flavonoid fraction at a dose of 100 mg/kg b.w.; PF50—diabetic mice treated with polar fraction at a dose of 50 mg/kg b.w.; PF100—diabetic mice treated with polar fraction at a dose of 100 mg/kg b.w.; STZMET—diabetic mice treated with Metformin (Sigma-Aldrich^®^—St. Louis, MO, USA) at a dose of 300 mg/kg b.w‥ Animals were treated orally by gavage once a day for 7 consecutive days according to the experimental group described above and at the end of treatment were submitted to intraperitoneal glucose tolerance test (ipGTT) according to topic “Intraperitoneal glucose tolerance test (ipGTT) described below”.

### Determination of lithospermoside (3) dosage by fasting glycemia and body weight monitoring

A different set of animals was randomly divided into six groups (n = 8/group): CTLSAL–non-diabetic control mice treated with vehicle (0.9% NaCl); STZSAL—diabetic mice treated with vehicle (0.9% NaCl); LITHOS1, LITHOS5, LITHOS10 and LITHOS20—diabetic mice treated with lithospermoside (**3**) at the doses of 1, 5, 10 and 20 mg/kg b.w., respectively. Animals were treated orally by gavage once a day for 14 consecutive days according to the experimental group. Mice weight and the fasting glycemia were measured weekly. The fasting glycemia was measured using a glucometer (One-touch, Johnson & Johnson). The dose that produced the highest decrease in blood glucose and least weight loss, *i*.*e*., the dose of 10 mg/kg b.w. of lithospermoside (**3**), was retained for further assays.

### Treatment with lithospermoside (3) for fasting glycemia and intraperitoneal glucose tolerance test (ipGTT) measurements

The animals were randomly divided into four groups (n = 8/group): CTLSAL—non-diabetic control mice treated with vehicle (0.9% NaCl); STZSAL—diabetic mice treated with vehicle (0.9% NaCl); LITHOS10—diabetic mice treated with lithospermoside (**3**) at a dose of 10 mg/kg b.w.; STZMET—diabetic mice treated with Metformin at a dose of 300 mg/kg b.w‥ Animals were treated orally by gavage once a day for 14 consecutive days according to the experimental group. Fasting glycemia was measured weekly using a glucometer (One-touch, Johnson & Johnson). At the end of treatment, animals were submitted to intraperitoneal glucose tolerance test (ipGTT) according to the topic “Intraperitoneal glucose tolerance test (ipGTT) described below”.

### Intraperitoneal glucose tolerance test (ipGTT)

At the end of the respective treatments (seven or fourteen days, described above), all the animal groups were submitted to the glucose tolerance test. Animals were deprived of food for 12-14h and fasting blood glucose was measured before an intraperitoneal load of D-glucose (2.0 g/kg b.w.) and defined as time 0; Subsequently the glucose load, blood samples were collected in the following two hours at 15, 30, 60, 90, and 120 min. The blood glucose was measured in all collected samples. Blood samples were obtained in tail tip under anesthesia procedures using Tiopental^®^ (Sigma-Aldrich^®^—St. Louis, MO, USA) (60 mg/kg b.w.) and glucose levels were measured using an enzymatic glucose detection kit (Dolles^®^, Goiás, Brazil).

### Statistical analysis

The results are expressed as means ± standard error of means (S.E.M.). Statistical analysis was performed using Instat 3® software. The symmetry of the data was tested by ShapiroWilk’s and Barllet normality tests. For multiple comparisons, ANOVA was used followed by Tukey´s post-test. The significance level adopted was p<0.05.

## Results

Our previous study demonstrated that the hydroalcoholic leaves extract of *B*. *holophylla* presented hypoglycemic effects in diabetic mice [3]. To identify the compounds responsible for the blood glucose-lowering effects, an in-depth phytochemical investigation of the extract was performed and enriched fractions with defined compositions were prepared for *in vivo* testing. This study was performed on an independent extract batch from the one used in our prospective *in vivo* investigation.

### Phytochemical investigation of the extract

#### Metabolite profiling of the extract and fractionation

**Fig 1:**
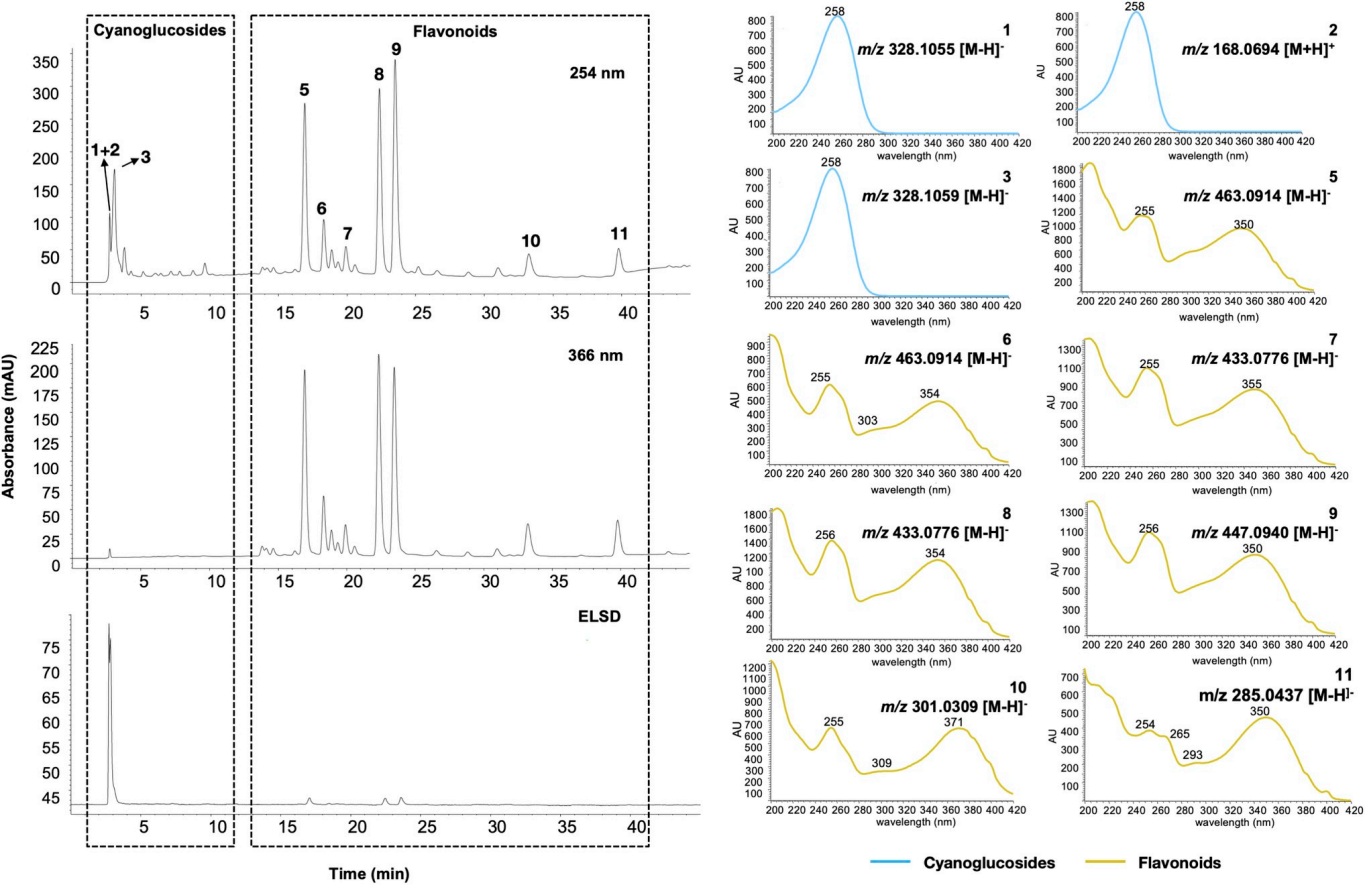
HPLC-PDA-ELSD analysis of the hydroalcoholic extract of *Bauhinia holophylla* leaves. Grey dark dashed rectangles represent the presence of cyanoglucoside derivatives (left) and the flavonoid derivatives (right) in the HPLC chromatogram. UV of each peak was obtained by HPLC-PDA analysis and molecular weight was determined by UHPLC-TOF-HRMS analysis.

To obtain a comprehensive survey of the phytochemical composition of the hydroalcoholic leaves extract, metabolite profiling was performed by High performance liquid chromatography coupled to photodiode array and evaporative light scattering detectors (HPLC-PDA-ELSD) and ultra-performance liquid chromatography coupled to time-of-flight high-resolution mass spectrometer (UHPLC-TOF-HRMS). The UV and HRMS data obtained suggested the presence of known flavonoids putatively identified by Camaforte and collaborators (Fig 1) [3]. However, for complete structure assignments, these compounds were isolated and identified as flavonoid aglycones and mono-glycosylated flavonoid derivatives (see below).

Besides, the ELSD chromatogram revealed the presence of polar compounds that were present in much higher amounts than flavonoids (*R*_*t*_ = 1–5 min in Fig 1). In the polar region of the chromatogram, compound **3**, detected at *R*_*t*_ of 0.58 min presented a molecular ion at *m/z* 328.1072 [M-H]^-^ corresponding to C_14_H_19_NO_8_. A cross-search of this molecular formula against all compounds previously isolated from *Bauhinia* species indicated that this compound could be lithospermoside, a non-cyanogenic cyanoglucoside previously described in *Bauhinia fassoglensis* Kotschy ex Schweinf. [16] or riachin, an isomer of lithospermoside previously described in *Bauhinia pentandra* [17].

### Preliminary fractionation of the active extract by vacuum liquid chromatography (VLC)

To localize the compounds responsible for the hypoglycemic activity of *B*. *holophylla*, two enriched fractions with complementary chemical composition were prepared in a large scale for *in vivo* evaluation using the intraperitoneal glucose tolerance test (ipGTT) [4]. For this, the hydroalcoholic leaves extract was first fractionated by vacuum liquid chromatography (VLC) using silica C_18_ as stationary phase (S2 Fig in S1 File) to rapidly obtain two fractions in large amounts compatible with further *in vivo* assay, one containing the polar compounds and other with the flavonoid derivatives. The HPLC-PDA analysis confirmed the presence of flavonoids (**5**–**11**) in the flavonoid fraction (FF), while the polar compounds (**1–3**) were present in the polar fraction (PF).

### Investigation of the hypoglycemic activity of *B*. *holophylla* extract and enriched fractions through the *in vivo* intraperitoneal glucose tolerance test (ipGTT)

**Fig 2:**
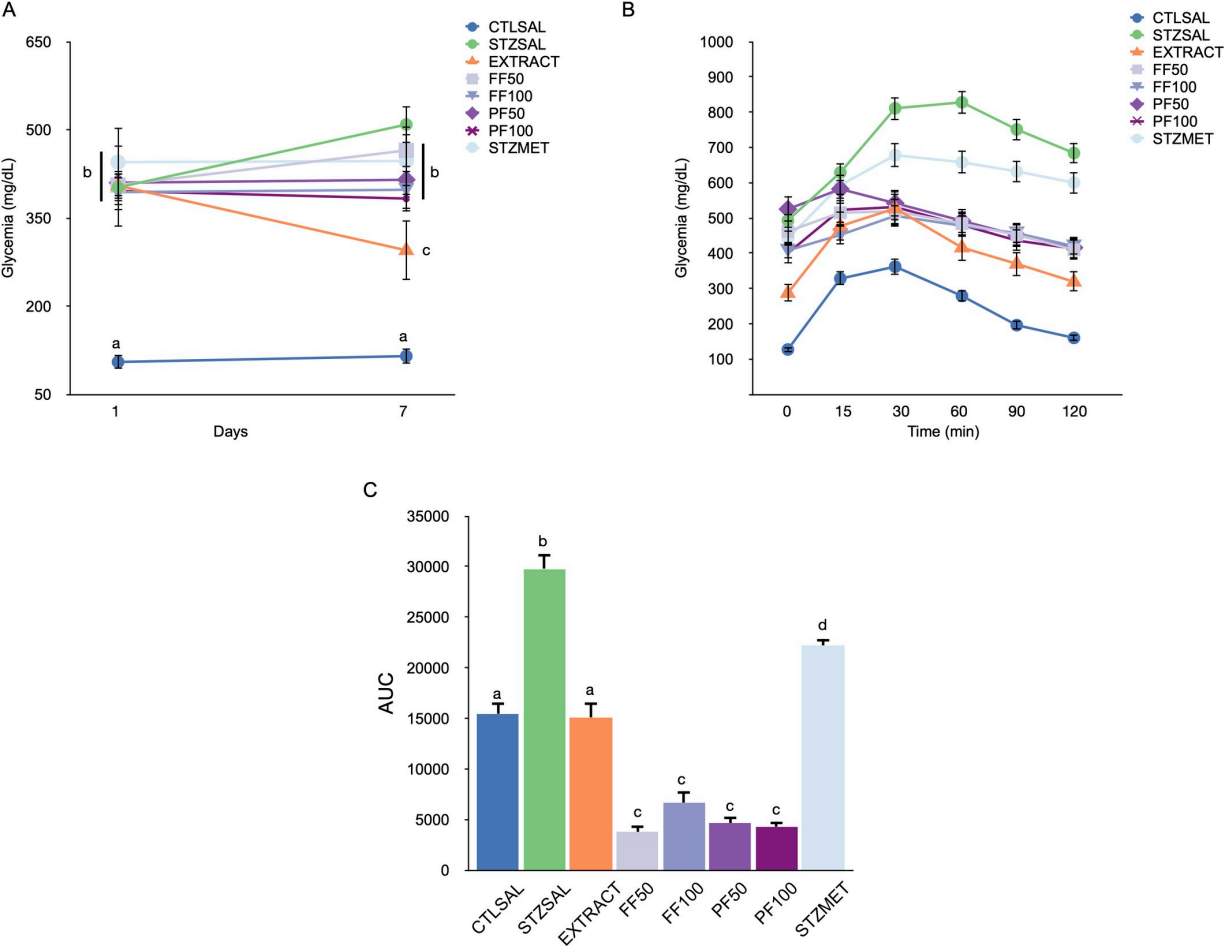
(**A**) Effect on blood glucose tolerance of treatment with the hydroalcoholic extract *of Bauhinia holophylla* leaves (400 mg/kg b.w.) and the flavonoid fraction (FF50) 50 mg/kg b.w., (FF100) 100 mg/kg b.w., polar fraction (PF50) 50 mg/kg b.w. and (PF100) 100 mg/kg b.w. and (**B**) area under the curve. The results are expressed as means ± SEM (n = 8/group). Different letters indicate significant differences between groups. ANOVA followed by Tukey-*post-test*, p<0.05.

Based on the doses determined in our previous study [3] the new extract batch was re-evaluated by the *in vivo* ipGTT at the optimal dose of 400 mg/kg b.w. and display the expected activity (Fig 2A and 2B and S1 Table in S1 File).

After diabetes induction, the STZSAL group presented an expected increase in fasting glycemia compared to CTLSAL. The treatment with hydroalcoholic leaves extract (EXTRACT—400 mg/kg b.w.) for 7 days led to a significant decrease by 40% (p<0.05) on the 7^th^ day in fasting blood glucose levels compared to the STZSAL. The treatment with the flavonoid fraction [6] at different doses; FF50 and FF100 (50 and 100 mg/kg b.w.) and the polar fraction (PF) at different doses; PF50 and PF100 (50 and 100 mg/kg b.w.) was not effective in reducing fasting blood glucose (Table 1).

**Table 1:** Effect on fasting blood glucose (mg/dl) of the treatment with hydroalcoholic extract of *Bauhinia holophylla* leaves (400 mg/kg b.w.), flavonoid fraction (FF50) 50 mg/kg b.w. and (FF100) 100 mg/kg b.w., polar fraction (PF50) 50 mg/kg b.w. and (PF100) 100 mg/kg b.w.

	CTLSAL	STZSAL	EXTRACT	FF50	FF100	PF50	PF100	STZMET
**DAY1**	111.2 ± 8.3^a^	401.3 ± 30.9^b^	410.5 ± 30.9^b^	401.0 ± 20.9^b^	399.9 ± 20.9^b^	415.0 ± 29.3^b^	420.7 ± 23.1^b^	441.8 ± 42.9^b^
**DAY7**	127.1 ± 9.4^a^	493.2 ± 36.3^b^	287.3 ± 35.7^c^	462.9 ± 25.5^b^	408.2 ± 26.4^b^	425.0 ± 28.4^b^	400.1 ± 25.0^b^	442.2 ± 32.1^b^

Results expressed as means ± SEM. Different letters indicate significant differences (ANOVA followed by Tukey’s *post-test*, n = 8, p < 0.05).

However, after 7 days of treatment, both enriched fractions and the EXTRACT (hydroalcoholic leaves extract) presented an improvement of glucose tolerance in the ipGTT (Fig 2A). The EXTRACT (400 mg/kg b.w.) and the FF and PF fractions (50 and 100 mg/kg b.w.) produced a progressive decrease in glycemia from 30 to 120 min after the glucose load.

The resulting area under the curve (AUC) showed a better improvement on glucose tolerance of the PF (50 and 100 mg/kg b.w.) and FF (50 and 100 mg/kg b.w.) compared with the EXTRACT (400 mg/kg b.w.), STZSAL and STZMET (p <0.0001) (Fig 2B). Nonetheless, no difference was observed among the different fractions.

In this test, metformin showed a lower effect than the crude extract and fractions. Metformin acts through the inhibition of hepatic gluconeogenesis by activating the 5´-adenosine monophosphate (AMP) kinase (AMPK) via a liver kinase B1 (LKB1) dependent mechanism. As we demonstrate [3], *B*. *holophylla* exerts the hypoglycemic action mainly through GSK3-beta inhibition and glycogenesis activation, which may be a more significant disfunction in this model of diabetes. Surprisingly there was no significant difference between the groups treated with flavonoid and polar fractions at the same concentration, suggesting that both fractions, having very different secondary metabolite compositions, presented similar biological activities.

Our preliminary ipGTT results with the hydroalcoholic leaves extract (EXTRACT) and the enriched fractions encouraged us to further investigate the active principles responsible for the hypoglycemic activity.

### Isolation of the main compounds found in the active fractions

**Fig 3:**
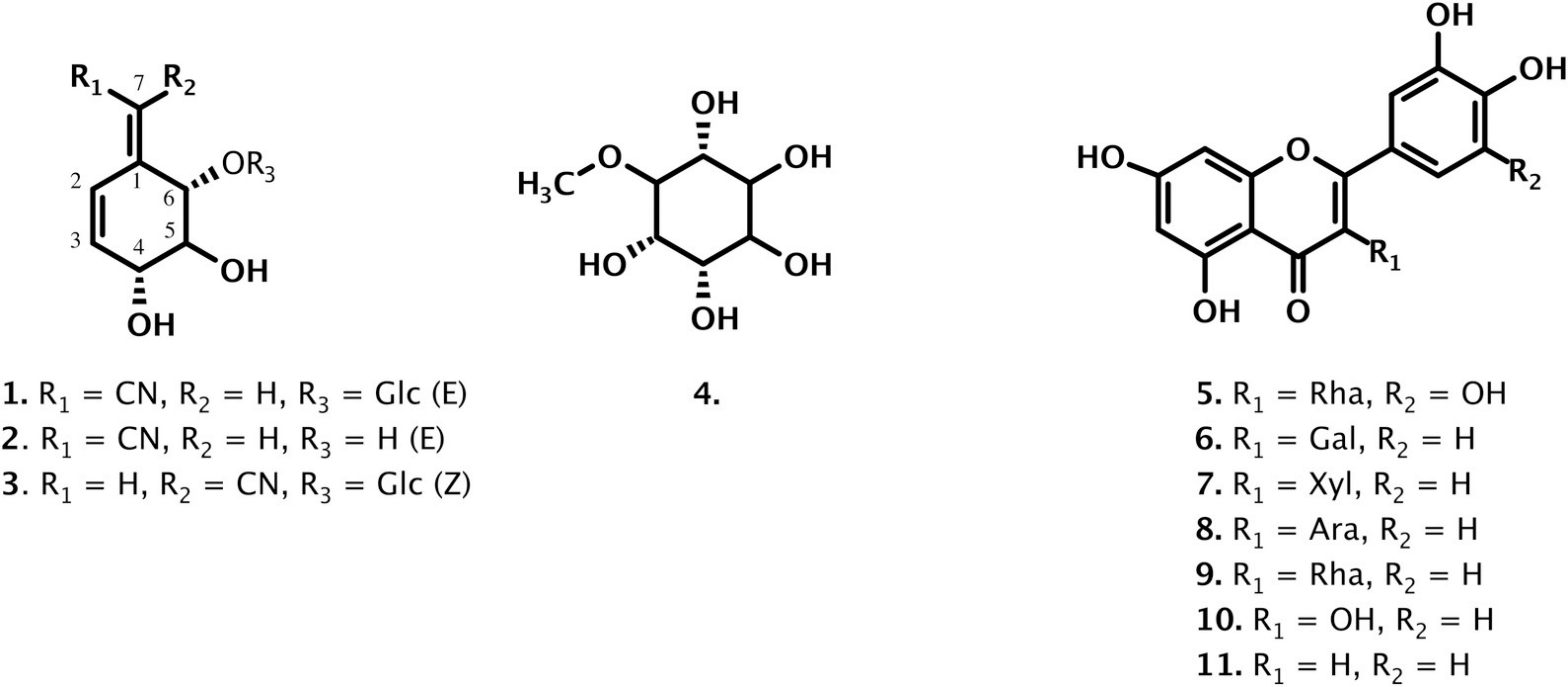
Isolated compounds from the hydroalcoholic leaves extract of *Bauhinia holophylla*.

For the identification of the active compounds, the hydroalcoholic leaves extract of *B*. *holophylla* was purified by medium pressure chromatography coupled to ultraviolet detector (MPLC-UV). The analytical HPLC conditions were first optimized and transferred to the MPLC with a gradient transfer method [10] (S3 Fig in S1 File). Using this approach, it was possible to obtain and identify four compounds (**1–3**, **7**) in one-step and the other seven compounds (**5**, **6**, **8**–**11**) with further semi-preparative HPLC-UV purification (Fig 3).

Their NMR analysis revealed the following flavonoids: myricitrin (**5**) [18], hyperin (**6**) [19], reynoutrin (**7**) [20], quercetin-3-O-*α*-*L*-arabinofuranoside (**8**) [20], quercitrin (**9**) [21], quercetin (**10**) [22], luteolin (**11**) [23] (S4-S48 Figs in S1 File). In the polar fraction the structure of the dereplicated cyanoglucosides were confirmed to be (-)-lithospermoside (**3**) [16, 24] and its steric isomer aglycone at C-1 (+)-(1E,4*R*,5*S*,6*S*)-4,5,6-trihydroxy-2-cyclohexen-1-ylideneacetonitrile (**2**) isolated for the first time in *Semiaquilegia adoxoides* [25]. In addition to these know compounds, an original stereoisomer of compound **3** was isolated and described below.

**Fig 4:**
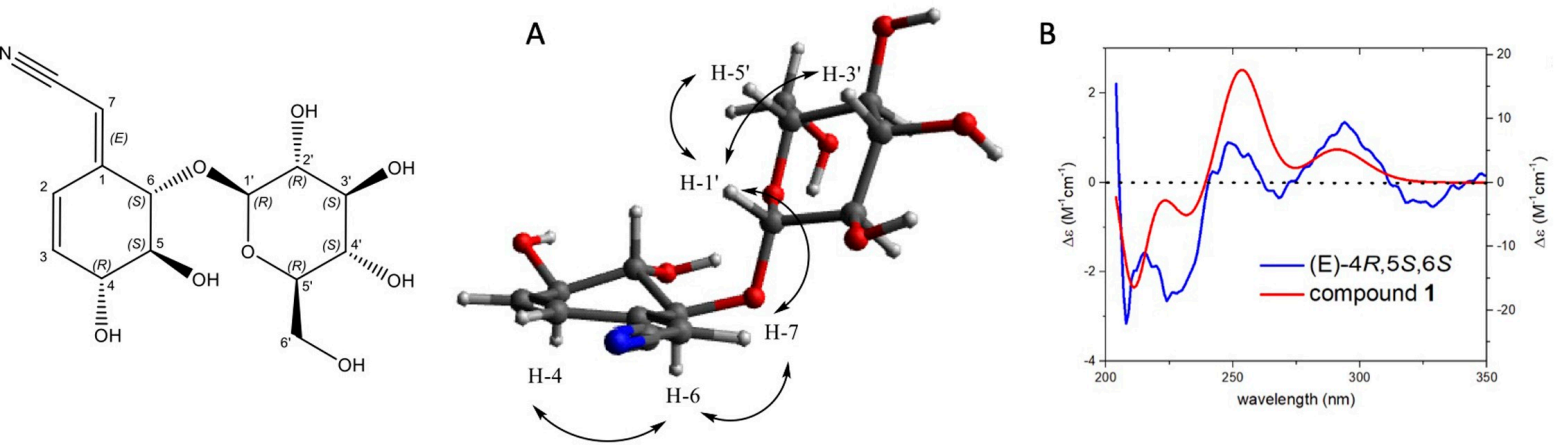
(**A**) NOESY correlations observed for compound **1**. (**B**) Experimental (red) and TDDFT calculated (blue) ECD spectra of enantiomer (E-4*R*, 5*S*, 6*S*) of compound **1** at the CAM-B3LYP/6-31G** level.

The ESI-HRMS spectrum of **1** showed the same molecular ion at *m/z* 328.1055 [M-H]^-^ as **3** and the NMR data confirmed that **1** was an isomer of lithospermoside (**3**). The large coupling constants between H-4 and H-5 (*J* = 8.3 Hz), and between H-5 and H-6 (*J* = 10.5 Hz) indicated that the configuration of the hydroxyls was the same as that of lithospermoside. The NOESY correlation between H-7 and H-6 indicated that the configuration of the double bond in C-1 was E like that of the aglycone (**2**) (Fig 4). The experimental ECD spectrum of **2** indicated two negative Cotton effects (CE) at 280 and 206 nm along with a positive CE at 227 nm, which could be due to π→π* transition of pentenenitrile and olefin groups. The TDDFT calculated ECD spectra for 4*R*,5*S*,6*S* stereoisomer showed a similar pattern with two negative CE at 280 and 205 nm and one positive CE around 230 nm (S1 Fig in S1 File). Compound **1** ((-)-(1E,4*R*,5*S*,6*S*)-6-(*β*-D-glucopyranosyloxy)-4,5-dihydroxy-2-cyclohexen-1-ylideneacetonitrile) was identified as the C-1 steric isomer of lithospermoside (**3**) and is described here for the first time. Also, the absolute configuration of **3** was established by ECD spectroscopy (S1 Fig in S1 File).

### Effects of lithospermoside (3) on body weight, fasting blood glucose and intraperitoneal glucose tolerance test (ipGTT)

Previous studies have demonstrated the effects of the flavonoids myricetin, quercetin and their respective glycoside derivatives in different *in vivo* models of diabetes [26–30] which explain the *in vivo* activity measured for the flavonoid fraction (FF).

On the other hand, the compounds found in the polar fraction which exhibited similar hypoglycemic activity have never been tested for such activity. Since lithospermoside (**3**) was the main cyanoglucoside in this fraction, the hypoglycemic potential of this compound was further investigated. As the treatment with the enriched fractions (FF and PF) for 7 days did not lead to a significant decrease in fasting blood glucose, although showed a better glucose handling on the GTT assay, the duration of the treatments with lithospermoside (**3**) was increased to 14 days.

The effects of lithospermoside (**3**) treatment at four different doses (1, 5, 10, and 20 mg/kg b.w.) for 14 days were evaluated on body weight and fasting blood glucose on diabetic mice. The doses range was estimated by quantification of lithospermoside (**3**) in the active hydroalcoholic leaves extract and polar fraction (S51-S54 Figs in S1 File). The quantification performed by ^1^H NMR estimated lithospermoside (**3**) at 1.7% w/w in the hydroalcoholic extract and 5.9% w/w in the polar fraction (PF). These percentages correspond to a range of 3.0 mg (estimated from 50 mg of the PF) to 6.8 mg (estimated from 400 mg of hydroalcoholic extract) of lithospermoside (**3**) (see experimental section). Based on this result, a dose range from 1 to 20 mg/kg b.w. was selected for performing the *in vivo* assay on fasting blood glucose.

**Fig 5:**
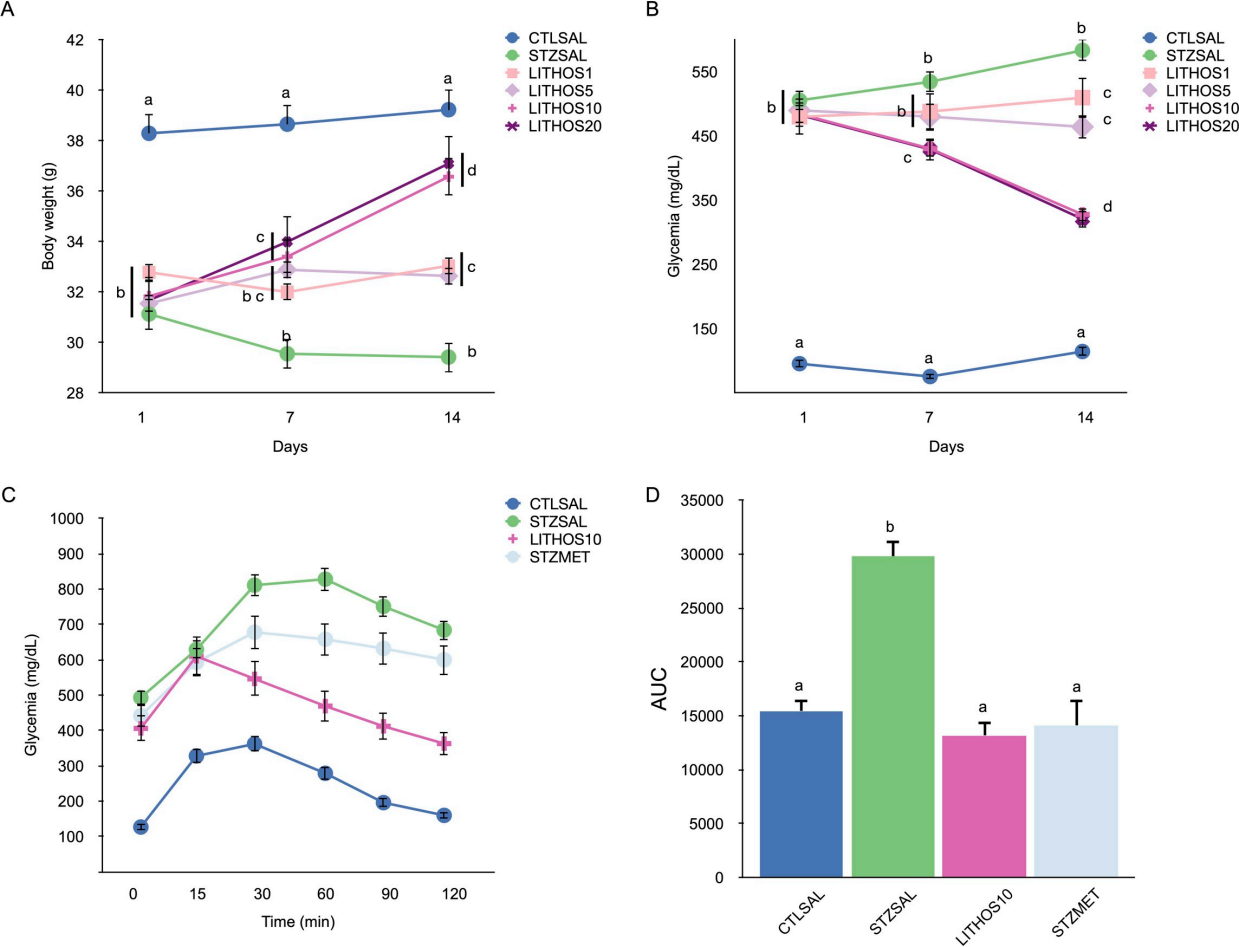
Effect of lithospermoside (**3**) at the doses of 1, 5, 10, and 20 mg/kg b.w. treatment on (**A**) body weight (**B**) on fasting blood glucose (**C**) at dose 10 mg/kg b.w. on glucose tolerance test and (**D**) area under the curve. The results are expressed as means±SEM (n = 10/group). Different letters indicate significant differences between groups. ANOVA followed by Tukey-*post-test*, p<0.05.

As shown in Fig 5A, the diabetic group (STZSAL) showed a significant decrease (p<0.05) in body weight compared to the control group (CTLSAL). Nevertheless, the diabetic group treated with lithospermoside (**3**) at 10 and 20 mg/kg b.w. (LITHOS10 and LITHOS20) showed a significant increase (p<0.05) in body weight compared to the STZSAL group after 7 and 14 days. The mice treated with lithospermoside (**3**) at 1 and 5 mg/kg b.w. doses (LITHOS1 and LITHOS5) maintained the body weight and presented a significant difference (p<0.05) after 14 days when compared with STZSAL. These results indicate that lithospermoside (**3**) prevents body weight loss and improves some of the most intuitive indicators of diabetes.

All diabetic groups (STZSAL and LITHOS) showed significantly higher fasting blood glucose than the CTLSAL group on the first day of the treatment (Fig 5B). However, the diabetic mice treated with lithospermoside (**3**) at 10 and 20 mg/kg b.w. (LITHOS10 and LITHOS20) showed a progressive decrease of glycemia by 34% (p<0.05) when compared to the STZSAL. The doses of lithospermoside (**3**) at 1 and 5 mg/kg b.w. (LITHOS1 and LITHOS5) did not alter the glycemia although there was a significant difference (p<0.05) when compared with the STZSAL group after 14 days of treatment.

Since 10 and 20 mg/kg b.w. of lithospermoside (**3**) produced similar glucose-lowering effects, the group treated with the dose of 10 mg/kg b.w. was selected to further investigate the effect of lithospermoside (**3**) on the ipGTT assay.

The ipGTT assay was thus performed after 14 days of treatment with lithospermoside (**3**) at dose of 10 mg/kg b.w. in comparison with metformin. The values obtained from the ipGTT showed, as expected, an increased fasting glycemia of the diabetic control group (STZSAL) at the time 0 in comparison with CTLSAL (Fig 5C). After the glucose injection, the STZSAL group had an increase in glucose levels, which was maintained during the experiment. On the other hand, the LITHOS10 group had a decrease in glucose levels from 30 min, showing an improvement of glucose tolerance when compared to STZSAL and STZMET. As expected, the area under curve showed that the glucose disappearance rate from blood was impaired in the STZSAL group when compared to CTLSAL (Fig 5D). However, STZMET and LITHOS10 groups showed an improvement in glucose tolerance when compared to STZSAL.

In this assay, the LITHOS10 group showed similar results when compared to the STZMET group. It has to be noted that the STZMET group received metformin (the most prescribed drug to treat diabetes) at doses 30 times higher (300 mg/kg b.w.) than those administrated for lithospermoside (**3**) (10 mg/kg b.w.).

### ^1^H NMR quantification of the major compounds

The proton nuclear magnetic resonance (^1^H NMR) quantification revealed that the hydroalcoholic leaves extract of *B*. *holophylla* contained 1.7% w/w of lithospermoside (**3**) and 3.1% w/w of total flavonoids (see experimental section). Surprisingly, the NMR quantification also revealed the presence of a large amount (9.5% w/w) of pinitol (3-*O*-methyl-chiroinositol) (**4**), a monosaccharide previously isolated from *Bougainvillea spectabilis*. Pinitol was already described for its *in vivo* antidiabetic properties [31, 32].

The ^1^H NMR quantitative analysis of the PF revealed 5.9% w/w of listhospermoside (**3**) and 3.7% w/w of pinitol (**4**). These percentages correspond to 3.0 and 5.9 mg of lithospermoside in the 50 and 100 mg/kg b.w. of the PF doses, respectively and to 1.9 and 3.7 mg of pinitol in the 50 and 100 mg/kg b.w. of the PF doses, respectively. The ^1^H NMR quantitative analysis of the FF revealed 27.5% w/w of the total amount of flavonoids, which corresponds to 13.8 and 27.5 mg in 50 and 100 mg of the flavonoid fraction doses.

To check the presence of these compounds in the traditional preparation, the leaves of *B*. *holophylla* were infused with water for 30 min. The water infusion extract was lyophilized and analyzed by HPLC-PDA. The HPLC analyses revealed that the water infusion and the hydroalcoholic extract exhibited similar metabolite profiles (S55 Fig in S1 File) for compounds other than sugars. As expected, the ^1^H NMR quantification of the infusion also revealed the presence of a significant amount of pinitol (**4**) (14.3%). The presence of overlapping signals in the NMR profile of the infusion did not permit a precise quantification of the other constituents.

## Discussion

The leaves of *Bauhinia holophylla* have long been used in traditional medicine in South America to treat diabetes [2]. However, the active principles responsible for the glucose-lowering effects were not described. In the present study, besides the mono-glycosylated flavonoids (3.1% w/w), the comprehensive chemical profiling of the active hydroalcoholic leaves extract revealed the presence of lithospermoside (**3**) (1.7%) and pinitol (**4**) (9.5% w/w).

The literature is very rich in describing the hypoglycemic effects of the flavonoids identified in the flavonoid enriched fraction and is summarized in several reviews [33, 34]. Several glycosylated flavonoids have been effective in reducing blood glucose levels through *in vivo* tests. Furthermore, flavonoid aglycone as quercetin, are important flavonoids with a wide range of pharmacological functions.

Pinitol (**4**), a monosaccharide previously isolated from *Bougainvillea spectabilis*, was already reported to possess antidiabetic properties [31, 32]. Treatment with pinitol was shown to be effective in ameliorating glucose and insulin tolerance as well as in reducing body weight loss in diabetic rats due to its insulin-like role [32, 35]. A recent review discusses a series of work on some molecules belonging to cyclitols, including *D*-pinitol, in the treatment of metabolic syndrome and diabetes [36]. Clearly, pinitol has insulin-like action by activating the glycolysis pathway. In particular, pinitol increases the activity of the glycolytic enzyme pyruvate kinase, as well as the phosphorylation of phosphoinositide 3-kinase/protein kinase B (PI3K/Akt) pathway enzymes, which are important in hepatic glucose metabolization. It is worth noting that these mechanisms of action correspond in parts, to the same anti-diabetic mechanism previously described for the *B*. *holophylla* extract in STZ-hyperglycemic animals [3]. In this study, the authors described several mechanisms by which the crude extract of *B*. *holophylla* leads to the decrease in blood glucose. The STZ-induced diabetic model used, produces hyperglycemia and hypoinsulinemia without the participation of inflammation or obesity. In this way, the effects of the extract are monitored in a “preserved” organism. It was observed that *B*. *holophylla* extract acts in reducing blood glucose in STZ-induced diabetic animals through hepatic mechanisms (especially by inhibiting GSK3-B and activating glycogenesis). Metformin also acts in the same processes, although there are still numerous doubts as to its mechanism of action. Thus, the choice of metformin as a positive control (or as a reference drug, better defined) in the present study, occurred in an attempt to observe the effects of substances of potentially similar action. This allowed us to compare our results with a drug widely prescribed as glucose-lowering due to its hepatic effects.

In this way, in the present study, using the same STZ-induced diabetic model, we observed a hypoglycemic action as well as an improvement of the glucose tolerance, following the treatment with the extract and the isolated compound. The diabetic mice treated with the extract (400 mg/kg b.w.) and the flavonoid and polar fractions (50 and 100 mg/kg b.w.) presented an improvement of glucose tolerance since it attenuated the elevation of the glycemia peak after glucose loading. A faster return to basal blood glucose values was also observed in these groups. However, only the extract decreased the fasting glycemia after 7 days of treatment in diabetic mice (Table 1). The mechanisms responsible for improving glucose handling after a glucose load needs to be investigated. Quantification data for the different groups of molecules with potential activity on diabetes indicated, when the daily doses are taken into account, that i) the amount of lithospermoside in the extract at 400 mg/kg b.w. and in the PF at 100 mg/kg b.w. are close (6.8 mg and 5.9 mg, respectively) and ii) the total flavonoid amount in the extract at 400 mg/kg b.w. and in the FF at 50 mg/kg b.w. is similar (12.4 mg and 13.8 mg, respectively). On the other hand, the amount of pinitol in the extract at 400 mg/kg b.w. (38.0 mg) was 10 times higher than in the PF at 100 mg/kg b.w. (3.7 mg) and pinitol was not present in the FF (S56 Fig in S1 File). This could explain the absence of a decrease in fasting glycemia in the groups treated with the enriched fractions PF and FF. The doses used, the time of treatment or the loss of synergistic action of the compounds could also be the cause of this decrease in activity.

Our results showed that isolated lithospermoside (**3**) is capable of preventing weight loss and reducing fasting blood glucose, as well as improving glucose tolerance at a dose of 10 mg/kg b.w (Fig 5). This dosage was estimated based on the NMR quantification of **3** in both extract and PF, which helped on the definition of the 1–20 mg/kg b.w. dose range used (see Results).

Our quantitative results also revealed for the extract that, pinitol (**4**) and to a less extent total flavonoids contributed to the antidiabetic activity in addition to lithospermoside (**3**).

It should be noted that a recent study using a different *in vivo* assay (oral glucose tolerance test—OGTT) suggested that the treatment with the infusion of *B*. *holophylla* (400 mg/kg b.w. doses, during 21 days) did not interfere with blood glucose levels of diabetic and non-diabetic rats [37]. The biological effects of the traditional infusion were not investigated in our study and levels of flavonoids, lithospermoside and pinitol were not assessed by Pinheiro et al. [37]. It is therefore not possible to explain these contradictory results. Nevertheless, such divergent results are not limited to this study. The review published by Cechinel (2009), on the biological potential of the genus reveals that several other species of *Bauhinia* have contradictory results on the effectiveness of diabetes treatment [1]. These data underline the need for in-depth studies of extracts from these species to quantify the level of the three classes of compounds that are involved in antidiabetic activity and to rationalize their use in traditional medicine.

Altogether, these results highlight the potent hypoglycemic action of lithospermoside on both fasting blood glucose and glucose tolerance test. To the best of our knowledge, this is the first report on the *in vivo* hypoglycemic properties of a cyanoglucoside derivative. This is relevant since, contrary to the other cyanoglucosides, lithospermoside is a non-cyanogenic natural product and thus seems likely to have probably no toxicity issues [38].

## Conclusion

Based on literature results and our data, it is possible to conclude that the hypoglycemic properties of the hydroalcoholic leaves extract of *B*. *holophylla* seem to result of the action of three class of compounds: the glycosylated derivatives of the flavonoids quercetin and myricetin, the polyol pinitol (**4**) and the non-cyanogenic cyanoglucoside lithospermoside (**3**). Further trials using different *in vivo* models for the study of diabetes will need to be conducted with lithospermoside to better understand its mechanism of action. In addition, in-depth synergistic studies to better understand the activity of the extract would be ideal. This would provide a sound basis for rational quality control of *Bauhinia* herbal preparations in the treatment of diabetes.

## Supporting information

S1 File (DOCX)
