# Supplementary material for: Hypoglycemic active principles from the leaves of Bauhinia holophylla: Comprehensive phytochemical characterization and in vivo activity profile
Source: PLoS One. 2021 Sep 24;16(9):e0258016. doi: 10.1371/journal.pone.0258016 (PMC8462688; doi:10.1371/journal.pone.0258016)
Supplement: S1 File — (DOCX) [file pone.0258016.s001.docx]

**Hypoglycemic active principles from the leaves of *Bauhinia holophylla*: comprehensive phytochemical characterization and *in vivo* activity profile.**

Luiz Leonardo Saldanha^1,2,3^, Aislan Quintiliano Delgado^1^, Laurence Marcourt^2,3^, Nathalia Aparecida de Paula Camaforte^1^, Priscilla Maria Ponce Vareda^1^, Samad Nejad Ebrahimi^4^, Wagner Vilegas^5^, Anne Lígia Dokkedal^1^, Emerson Ferreira Queiroz^2,3¶*^, Jean-Luc Wolfender^2,3¶*^ and José Roberto Bosqueiro^1¶*^

^1^ Faculty of Sciences, São Paulo State University (UNESP), CEP 17033-360, Bauru, São Paulo, Brazil.

^2^ School of Pharmaceutical Sciences, University of Geneva, CMU - Rue Michel-Servet 1, CH-1211 Geneva 4, Switzerland.

^3^ Institute of Pharmaceutical Sciences of Western Switzerland (ISPSO), University of Geneva, CMU - Rue Michel Servet 1, CH-1211 Geneva 4, Switzerland.

^4^ Department of Phytochemistry, Medicinal Plants and Drugs Research Institute, Shahid Beheshti University, G. C., Evin, Tehran, Iran.

^5^ Institute of Biociences, São Paulo State University (UNESP), Coastal Campus, CEP 11330-900, São Vicente, São Paulo, Brazil.

*** Corresponding Authors**

E-mail: emerson.ferreira@unige.ch (EFQ)

E-mail: jean-Luc.wolfender@unige.ch (JLW)

E-mail: jose.bosqueiro@unesp.br (JRB)

^¶^These authors equally contribute to this work as last authors


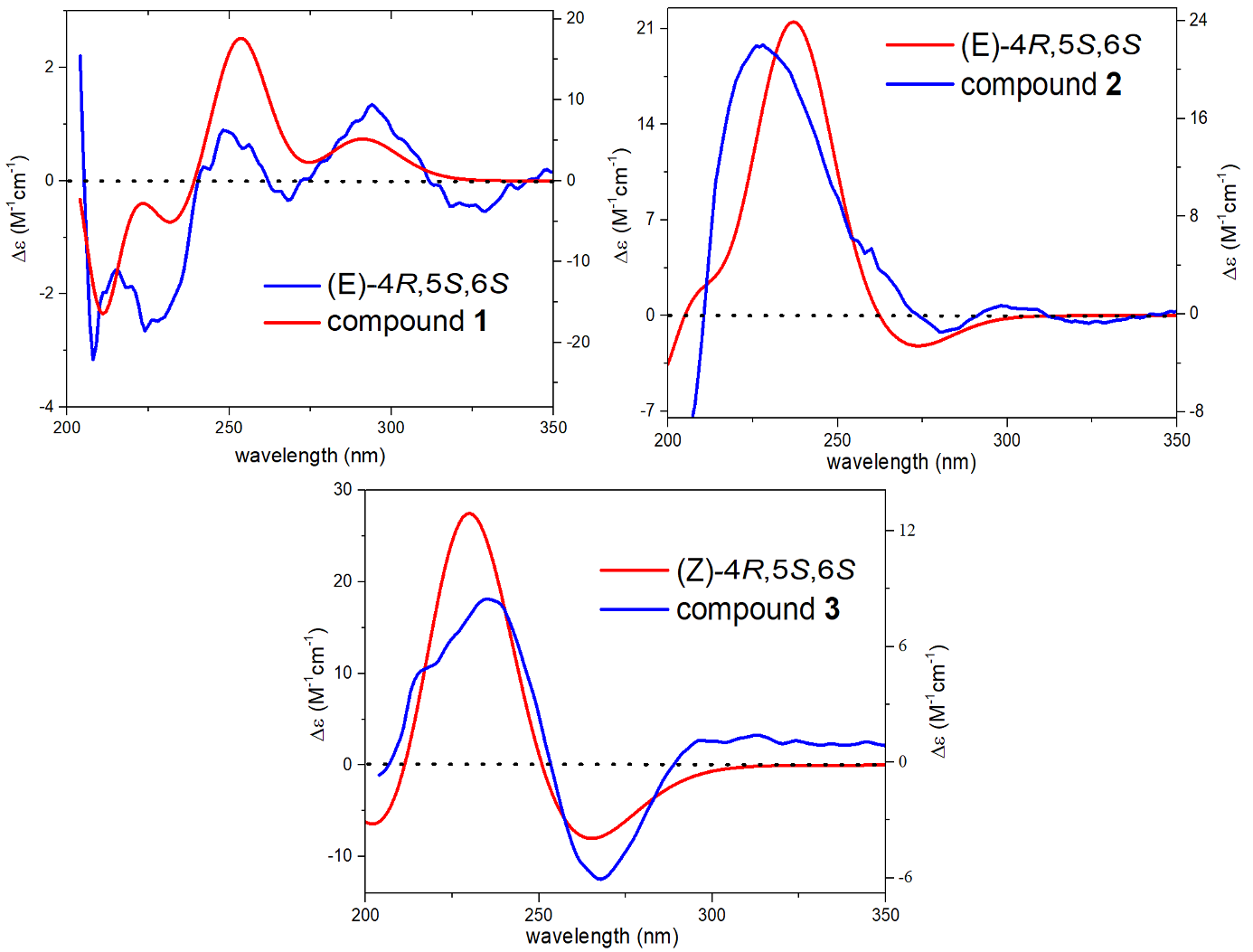


**S1 Fig**. Comparison of experimental ECD spectra with those of TDDFTs calculated for appropriate stereoisomer of compounds **1**-**3**. The calculations were performed with TDDFT at the cam-B3LYP/6-31G** level with MeOH as a solvent.

**
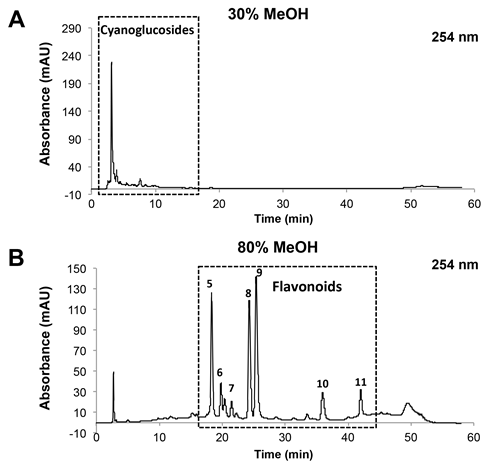
**

**S2 Fig.** HPLC-PDA analysis of the VLC fractions obtained from the hydroalcoholic leaves extract of *Bauhinia holophylla*. A) 30% MeOH VLC fraction. B) 80% MeOH VLC fraction.

**S1 Tab**. Effect on blood glucose tolerance of the treatment with hydroalcoholic extract *of Bauhinia holophylla* leaves (400 mg/kg b.w.) and the flavonoid fraction (FF50) 50 mg/kg b.w., (FF100) 100 mg/kg b.w., polar fraction (PF50) 50 mg/kg b.w. and (PF100) 100 mg/kg b.w.

| **Time (min)** | **CTLSAL** | **STZSAL** | **EXTRACT** | **FF50** | **FF100** | **PF50** | **PF100** | **STZMET** |
| --- | --- | --- | --- | --- | --- | --- | --- | --- |
| 0 | 127.0 ± 9.45 | 493.8 ± 36.37 | 287.3 ± 37.50 | 462.9 ± 27.55 | 408.2 ± 26.47 | 525.5 ± 28.43 | 400.1 ± 24.05 | 442.0 ± 32.15 |
| 15 | 328.1 ± 24.10 | 629.0 ± 55.47 | 477.1 ± 74.40 | 515.2 ± 26.45 | 452.6 ± 27.07 | 582.9 ± 37.74 | 523.5 ± 21.94 | 593.4 ± 31.77 |
| 30 | 362.3 ± 22.55 | 811.8 ± 53.06 | 527.5 ± 82.59 | 519.4 ± 30.48 | 506.3 ± 23.44 | 542.1 ± 20.93 | 531.5 ± 17.22 | 678.1 ± 41.83 |
| 60 | 279.5 ± 23.50 | 828.7 ± 48.76 | 416.8 ± 35.45 | 484.0 ± 38.04 | 478.1 ± 17.04 | 492.7 ± 21.09 | 480.1 ± 22.93 | 658.6 ± 44.51 |
| 90 | 196.0 ± 26.84 | 751.0 ± 52.43 | 370.7 ± 36.99 | 452.3 ± 40.78 | 457.8 ± 16.21 | 451.0 ± 25.85 | 436.5 ± 25.14 | 632.7 ± 39.71 |
| 120 | 160.1 ± 25.72 | 684.4 ± 66.32 | 319.0 ± 38.61 | 410.8 ± 45.89 | 420.1 ± 21.18 | 416.8 ± 23.63 | 413.3 ± 27.73 | 600.5 ± 38.51 |

Results expressed as means ± SEM (n = 8/group).


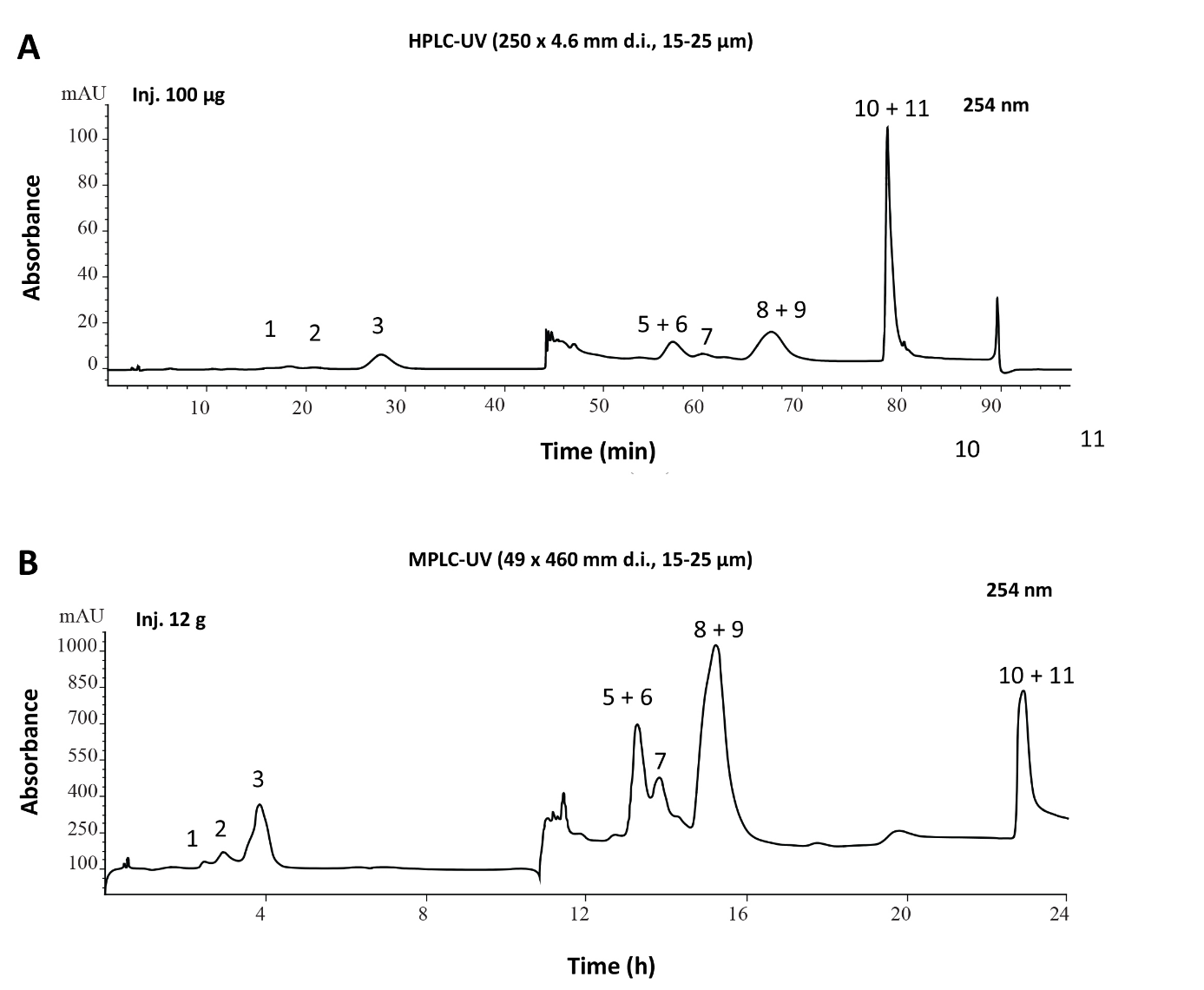


**S3 Fig.** Gradient transfer from HPLC to MPLC. A) HPLC-PDA analysis of the 70% EtOH leaves extract of *Bauhinia holophylla*. B) Preparative MPLC-UV chromatogram with the same extract.

**S4 Fig.** ^1^H NMR spectrum of compound **1** in D_2_O at 499.9 MHz.

**S5 Fig.** COSY NMR spectrum of compound **1** in D_2_O.

**S6 Fig.** Edited-HSQC NMR spectrum of compound **1** in D_2_O.

**S7 Fig.** HMBC NMR spectrum of compound **1** in D_2_O.

**S8 Fig.** NOESY NMR spectrum of compound **1** in D_2_O.

**S9 Fig.** ^1^H NMR spectrum of compound **2** in D_2_O at 499.9 MHz.

**S10 Fig.** COSY NMR spectrum of compound **2** in D_2_O.

**S11 Fig.** ^13^C-DEPTQ NMR spectrum of compound **2** in D_2_O at 126 MHz.

**S12 Fig.** Edited-HSQC NMR spectrum of compound **2** in D_2_O.

**S13 Fig.** HMBC NMR spectrum of compound **2** in D_2_O.

**S14 Fig.** NOESY NMR spectrum of compound **2** in D_2_O.

**S15 Fig.** ^1^H NMR spectrum of compound **3** in D_2_O at 499.9 MHz.

**S16 Fig.** COSY NMR spectrum of compound **3** in D_2_O.

**S17 Fig.** Edited-HSQC NMR spectrum of compound **3** in D_2_O.

**S18 Fig.** HMBC NMR spectrum of compound **3** in D_2_O.

**S19 Fig.** ^1^H NMR spectrum of compound **4** in D_2_O at 499.9 MHz.

**S20 Fig.** COSY NMR spectrum of compound **4** in D_2_O.

**S21 Fig.** Edited-HSQC NMR spectrum of compound **4** in D_2_O.

**S22 Fig.** HMBC NMR spectrum of compound **4** in D_2_O.

**S23 Fig.** ^1^H NMR spectrum of compound **5** in DMSO-*d*_6_ at 499.3 MHz.

**S24 Fig.** COSY NMR spectrum of compound **5** in DMSO-*d*_6_.

**S25 Fig.** ^13^C NMR spectrum of compound **5** in DMSO-*d*_6_ at 125.6 MHz.

**S26 Fig.** HMQC NMR spectrum of compound **5** in DMSO-*d*_6_.

**S27 Fig.** HMBC NMR spectrum of compound **5** in DMSO-*d*_6_.

**S28 Fig.** ^1^H NMR spectrum of compound **6** in DMSO-*d*_6_ at 499.3 MHz.

**S29 Fig.** COSY NMR spectrum of compound **6** in DMSO-*d*_6_.

**S30 Fig.** ^13^C NMR spectrum of compound **6** in DMSO-*d*_6_ at 125.6 MHz.

**S31 Fig.** HMQC NMR spectrum of compound **6** in DMSO-*d*_6_.

**S32 Fig.** HMBC NMR spectrum of compound **6** in DMSO-*d*_6_.

**S33 Fig.** ^1^H NMR spectrum of compound **7** in DMSO-*d*_6_ at 499.3 MHz.

**S34 Fig.** COSY NMR spectrum of compound **7** in DMSO-*d*_6_.

**S35 Fig.** HMQC NMR spectrum of compound **7** in DMSO-*d*_6_.

**S36 Fig.** HMBC NMR spectrum of compound **6** in DMSO-*d*_6_.

**S37 Fig.** ^1^H NMR spectrum of compound **8** in DMSO-*d*_6_ at 499.3 MHz.

**S38 Fig.** COSY NMR spectrum of compound **8** in DMSO-*d*_6_.

**S39 Fig.** ^13^C NMR spectrum of compound **8** in DMSO-*d*_6_ at 125.6 MHz.

**S40 Fig.** HMQC NMR spectrum of compound **8** in DMSO-*d*_6_.

**S41 Fig.** HMBC NMR spectrum of compound **8** in DMSO-*d*_6_.

**S42 Fig.** ^1^H NMR spectrum of compound **9** in DMSO-*d*_6_ at 499.3 MHz.

**S43 Fig.** COSY NMR spectrum of compound **9** in DMSO-*d*_6_.

**S44 Fig.** ^13^C NMR spectrum of compound **9** in DMSO-*d*_6_ at 125.6 MHz.

**S45 Fig.** HSQC NMR spectrum of compound **9** in DMSO-*d*_6_.

**S46 Fig.** HMBC NMR spectrum of compound **9** in DMSO-*d*_6_.

**S47 Fig.** ^1^H NMR spectrum of compound **10** in CD_3_OD at 499.3 MHz.

**S48 Fig.** ^13^C-DEPTQ NMR spectrum of compound **10** in CD_3_OD at 126 MHz

**S49 Fig.** ^1^H NMR spectrum of compound **11** in DMSO-*d*_6_ at 499.3 MHz.

**S50 Fig.** ^13^C-DEPTQ NMR spectrum of compound **11** in DMSO-*d*_6_ at 126 MHz.

**S51 Fig.** ^1^H NMR spectrum of 10 mg of hydroalcoholic leaves extract in 600 μL of a deuterated phosphate buffer (pH 7) containing 3.87 mM of TSP. The blue areas indicate the signals used for the quantification of lithospermoside and pinitol as well as the TSP at δ_H_ 0 used as internal standard.

**S52 Fig.** ^1^H NMR spectrum of 0.9 mg of flavonoid fraction in 600 μL of D_2_O containing 0.9 mM of TSP. The blue areas indicate the signals used for the quantification of flavonoids as well as the TSP at δ_H_ 0 used as internal standard.

**S53 Fig.** ^1^H NMR spectrum of 1.5 mg of polar fraction in 600 μL of D_2_O containing 0.9 mM of TSP. The blue areas indicate the signals used for the quantification of lithospermoside and pinitol as well as the TSP at δ_H_ 0 used as internal standard.

**S54 Fig.** ^1^H NMR spectrum of 4.8 mg of traditional water infusion in 600 μL of a deuterated phosphate buffer (pH 7) containing 5.805 mM of TSP. The blue areas indicate the signals used for the quantification of pinitol as well as the TSP at δ_H_ 0 used as internal standard.

**S55 Fig.** HPLC-PDA analysis of the hydroalcoholic leaves extract (A) and the traditional water infusion (B) of *Bauhinia holophylla*. HPLC conditions: X-Bridge C_18_ column (250 × 4.6 mm i.d., 5 μm, Waters, Milford, MA, USA), solvent system MeOH (B) and H_2_O (A), both containing 0.1% FA. The separation was performed on gradient mode as follow: 60% B to 100% of B in 60 min. Flow rate 1 mL/min; injection volume 10 μL; sample concentration 10 mg/mL in Methanol.

**S56 Fig**. Overview of the fractionation of the hydroalcoholic extract and the ^1^H NMR quantitation of lithospermoside (**3**), total flavonoids and pinitol (**4**).
